# Case Report: Efgartigimod: a beacon of hope for overlapping stiff-person syndrome and myasthenia gravis following thymoma

**DOI:** 10.3389/fimmu.2026.1825974

**Published:** 2026-07-20

**Authors:** Jingjing Nie, Weifan Yin

**Affiliations:** 1 Department of Neurology, The Second Xiangya Hospital, Central South University, Changsha, Hunan, China; 2 Clinical Medical Research Center for Stroke Prevention and Treatment of Hunan Province, Department of Neurology, The Second Xiangya Hospital, Central South University, Changsha, Hunan, China; 3 The “Double-First Class” Application Characteristic Discipline of Hunan Province, Clinical Medicine, Changsha Medical University, Changsha, Hunan, China

**Keywords:** autoimmune neurological disorders, efgartigimod, GAD65, myasthenia gravis, stiff-person syndrome, thymoma

## Abstract

The coexistence of stiff person syndrome (SPS) and myasthenia gravis (MG) is exceptionally rare, with fewer than 20 cases reported to date. Thymoma has been identified in a substantial proportion of these patients, suggesting a potential shared autoimmune mechanism. We report a 43-year-old woman who initially presented with progressive limb stiffness, painful muscle spasms, and gait impairment. She was diagnosed with SPS based on positive anti-glutamic acid decarboxylase (GAD) antibodies and characteristic electromyographic findings. Her symptoms improved after immunotherapy but relapsed following corticosteroid withdrawal. Four years later, she developed fluctuating limb weakness and ptosis and was subsequently diagnosed with anti-acetylcholine receptor antibody-positive MG associated with type B2 thymoma. Following thymectomy and postoperative radiotherapy, COVID-19 infection precipitated a myasthenic crisis requiring intensive care support. Conventional treatments, including plasma exchange, intravenous immunoglobulin, corticosteroids, and rituximab, failed to achieve satisfactory clinical improvement. In contrast, treatment with efgartigimod resulted in marked recovery of respiratory and motor function. During follow-up through December 2025, no recurrence of SPS symptoms or MG exacerbation was observed. This case highlights the complex association among SPS, MG, and thymoma and supports the possibility that thymoma may contribute to the development of multiple autoimmune neurological disorders. Furthermore, efgartigimod may represent a promising therapeutic option for refractory thymoma-associated MG in patients with overlapping SPS and MG. Long-term surveillance for thymoma and additional autoimmune manifestations should be considered in patients with SPS.

## Introduction

Stiff person syndrome (SPS) is a rare autoimmune neurological disorder characterized by progressive muscle stiffness and varying degrees of disability. Typical clinical manifestations include painful muscle spasms, chronic muscle pain, and recurrent falls (1). The pathogenesis of SPS is believed to involve autoantibodies against glutamic acid decarboxylase (GAD), glycine receptor α1 (GlyRα1), or the γ-aminobutyric acid (GABA) receptor-associated protein gephyrin, resulting in impaired inhibitory neurotransmission and increased neuromuscular excitability (2).

SPS is frequently associated with other autoimmune disorders, including type 1 diabetes (3), and autoimmune thyroiditis (4), as well as autoimmune neurological diseases, such as anti-LGI1 encephalitis (5),and more rarely, myasthenia gravis (MG) (6, 7). These overlapping autoimmune conditions often increase the complexity of diagnosis and treatment.

Myasthenia Gravis (MG) is an autoimmune neuromuscular disorder characterized by fluctuating weakness of skeletal muscles (8). MG is often associated with thymus abnormalities, including thymoma or thymic hyperplasia. Thymectomy is recommended as a vital treatment for thymoma associated MG (9). However, some patients may experience new-onset MG or worsening of existing MG after surgery (10). Around 8.5%-14.8% of myasthenia gravis (MG) patients are drug-refractory (11). This status is linked to early-onset MG, female gender, anti-MuSK antibodies, and thymoma (12). Factors like overwork, malnutrition, infections, insomnia, and cognitive or emotional disorders often contribute to treatment resistance (13).

The overlapping of SPS with MG is extremely rare, which were no more than twenty cases ever been reported (14). Herein, we report a patient who developed MG and type B2 thymoma four years after the onset of SPS. Following thymectomy, MG symptoms progressed after COVID-19 infection and proved refractory to plasma exchange, corticosteroids, and rituximab. Ultimately, periodic efgartigimod therapy resulted in marked clinical improvement, and the patient achieved minimal symptom expression within one month. In addition, we review previously reported cases of SPS coexisting with MG and summarize their clinical features, management, and prognosis. This case highlights the importance of long-term surveillance for thymoma and additional autoimmune neurological disorders in patients with SPS.

## Case description

### First stage

On January 15, 2018, a 43-year-old woman was admitted to the hospital with a one-month history of gradually progressive back pain and limb stiffness. She was previously healthy. By the time of presentation, she was already experiencing significant difficulty walking. On examination, her mental status, speech, and cranial nerves were unremarkable. Neurological examination revealed normal muscle bulk with increased tone in the flexors and extensors of the knees and ankles. Muscle strength and coordination were intact, and deep tendon reflexes were brisk. There was no evidence of fatigable muscle weakness. Sensory examination was normal. Serum anti-GAD65 antibody testing by cell-based assay yielded a positive result at a dilution of 1:100. but CSF anti-GAD65 antibody was negative. Routine laboratory investigations, including full blood count, vitamin B_12_, folate, thyroid and liver function tests, urea, electrolytes, glucose, cortisol, immunoglobulins, and serum protein electrophoresis, were unremarkable. Autoantibody screening for antinuclear, smooth muscle, mitochondrial, parietal cell, gliadin, reticulin, microsomal, thyroid peroxidase, and antineutrophil cytoplasmic antibodies was negative. Additionally, no antibodies against encephalomyelitis/encephalitis-associated antigens (NMDA, AMPA1, AMPA2, LGI1, CASPR2, GABABR, DPPX, IgLON5, GlyRα1, mGluR1, mGluR5, D2R, Neurexin-3a) or gangliosides (GM1, GM2, GM3, GM4, GD1a, GD1b, GD2, GD3, GQ1b, GT1a, GT1b, sulfatide) and were detected in either serum or CSF. Needle electromyography (EMG) demonstrated continuous motor unit activity (CMUA) in both agonist and antagonist muscles of the lower limbs, with superimposed bursts triggered by tactile stimulation. This abnormal activity was abolished following diazepam administration. No myotonic discharges, fasciculation potentials, or neuromyotonic discharges were observed. Electroencephalography and chest computed tomography (CT) revealed no abnormalities. The patient was treated with a five-day course of intravenous immunoglobulin (IVIG) and methylprednisolone pulse therapy, followed by oral prednisolone with gradual tapering. Clonazepam and eperisone were administered for symptomatic relief. The patient’s symptoms improved substantially after treatment. Based on the characteristic clinical presentation, positive anti-GAD65 antibodies, and electrophysiological findings, the patient fulfilled both the major and minor diagnostic criteria for SPS as defined by Baizabal-Carvallo et al. (15) However, in August 2018, shortly after discontinuation of prednisolone, she experienced a relapse characterized by worsening limb stiffness, dizziness, gait instability, and impaired balance. Repeat serum testing showed that anti-GAD65 antibodies remained positive. The patient was readmitted and treated with IVIG and prednisolone, resulting in gradual clinical improvement.

### Second stage

In March 2022, the patient developed fluctuating limb weakness and intermittent ptosis, which first appeared after COVID-19 vaccination. As these symptoms progressively worsened, she underwent further evaluation in October 2022. Neurological examination revealed fatigable weakness involving ocular and limb muscles. Serum anti-acetylcholine receptor (AChR) and anti-titin antibodies were positive, with anti-AChR antibody titer 29 nmol/L (ELISA; normal <0.45 nmol/L) and anti-titin antibody titer 3.76 U/mL (ELISA; normal <1.00 U/mL). Repetitive nerve stimulation demonstrated a significant decremental response, consistent with a postsynaptic neuromuscular transmission defect. Based on the clinical presentation, serological findings, and electrophysiological evidence, a diagnosis of MG was established. At diagnosis, the patient’s MG-ADL score was 7, the QMG score was 16, and the disease was classified as MGFA class MGFA IIA. Chest computed tomography revealed an anterior mediastinal mass suggestive of thymoma. Pyridostigmine treatment was initiated, and two sessions of plasma exchange were performed, resulting in symptomatic improvement. Following plasma exchange, the MG-ADL score improved from 7 to 2, and the QMG score improved from 16 to 8. The patient subsequently underwent thymectomy, and postoperative histopathological examination confirmed a World Health Organization (WHO) type B2 thymoma.

### Third stage

In February 2023, the patient completed postoperative radiotherapy. Later that month, she contracted COVID-19 and subsequently developed worsening fatigue, severe ptosis, generalized weakness, and progressive respiratory distress, in the absence of back pain, limb pain, or stiffness. At admission in March 2023, the patient presented with an MG-ADL score of 16, a QMG score of 28, and a serum anti-AChR antibody titer of 55.28 nmol/L. The disease rapidly progressed to myasthenic crisis (MGFA class V), necessitating non-invasive ventilation and transfer to the intensive care unit (ICU). During hospitalization, the patient underwent two sessions of plasma exchange followed by intravenous immunoglobulin (0.4 g/kg/day for 5 days), without significant clinical improvement. High-dose intravenous methylprednisolone (1 g/day for 3 days) was administered, followed by gradual dose tapering. Given the patient’s concurrent infectious complications, and in consideration of minimizing the risk of infusion-related adverse reactions and immunosuppression, as well as previous reports on the efficacy of low-dose rituximab regimens (16), rituximab was administered at doses of 100 mg and 500 mg on April 9 and April 10, respectively. Despite these treatments, respiratory symptoms and limb weakness persisted. On April 15, the MG-ADL score remained 16 and the QMG score remained 28. Given the inadequate response to conventional immunotherapies, efgartigimod was initiated at a dose of 10 mg/kg weekly for four consecutive weeks. The patient reported marked improvement in respiratory function and muscle strength, allowing discontinuation of ventilatory support and restoration of independent ambulation. She expressed satisfaction with the treatment outcome, noting that the periodic efgartigimod regimen enabled her to return to daily activities and regain functional independence. Following the first cycle of efgartigimod treatment, the MG-ADL score decreased from 16 to 7, the QMG score decreased from 28 to 15, serum IgG level dropped to 4 g/L, and serum anti-AChR antibody titer declined to 26.21 nmol/L. By July 2023, the patient had achieved minimal symptom expression (MSE), with an MG-ADL score of 0 and a QMG score of 3.

**Figure 1:**
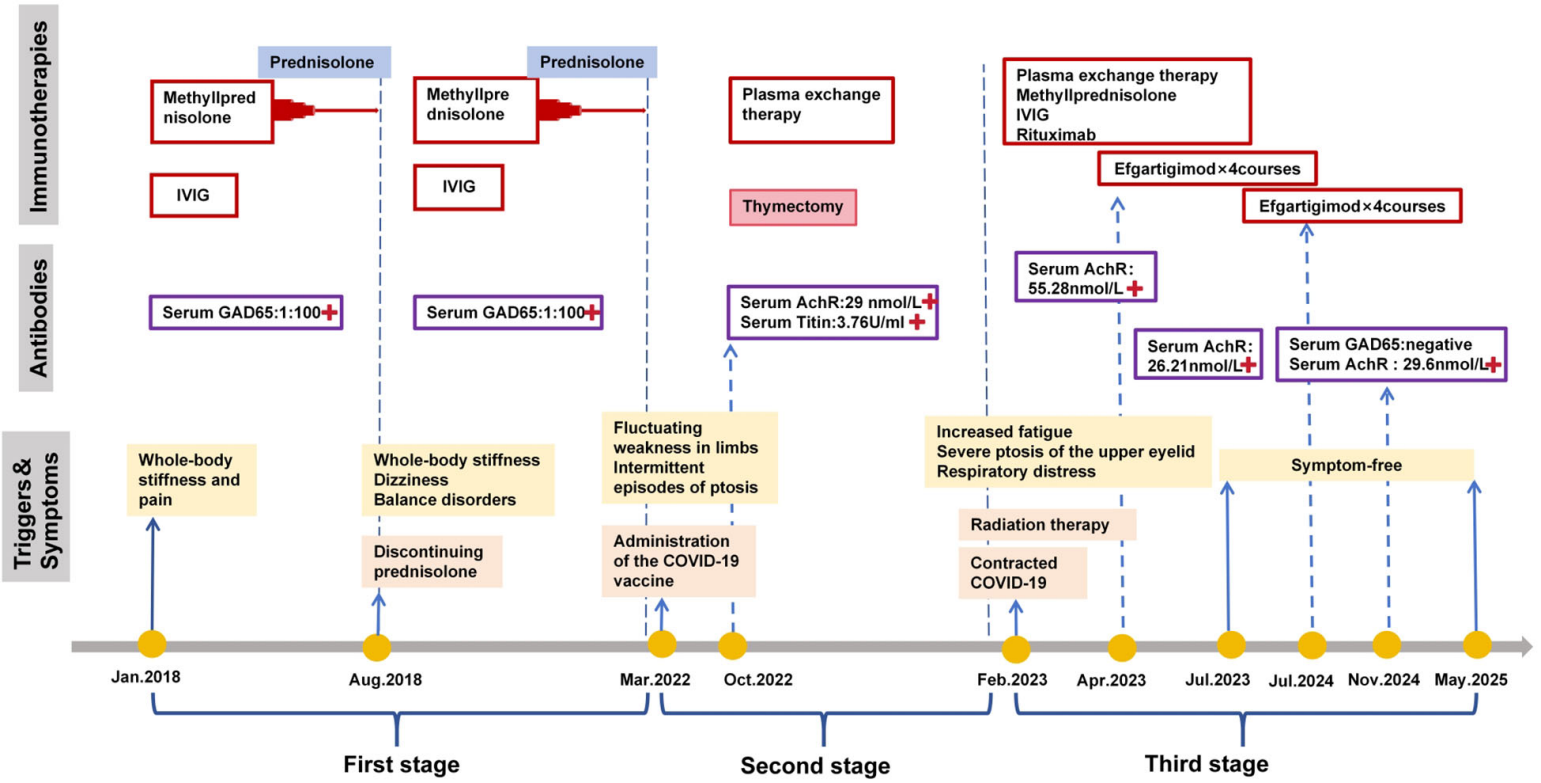
A timeline of events. +, positive; IVIG, intravenous immunoglobulin.

**Figure 2:**
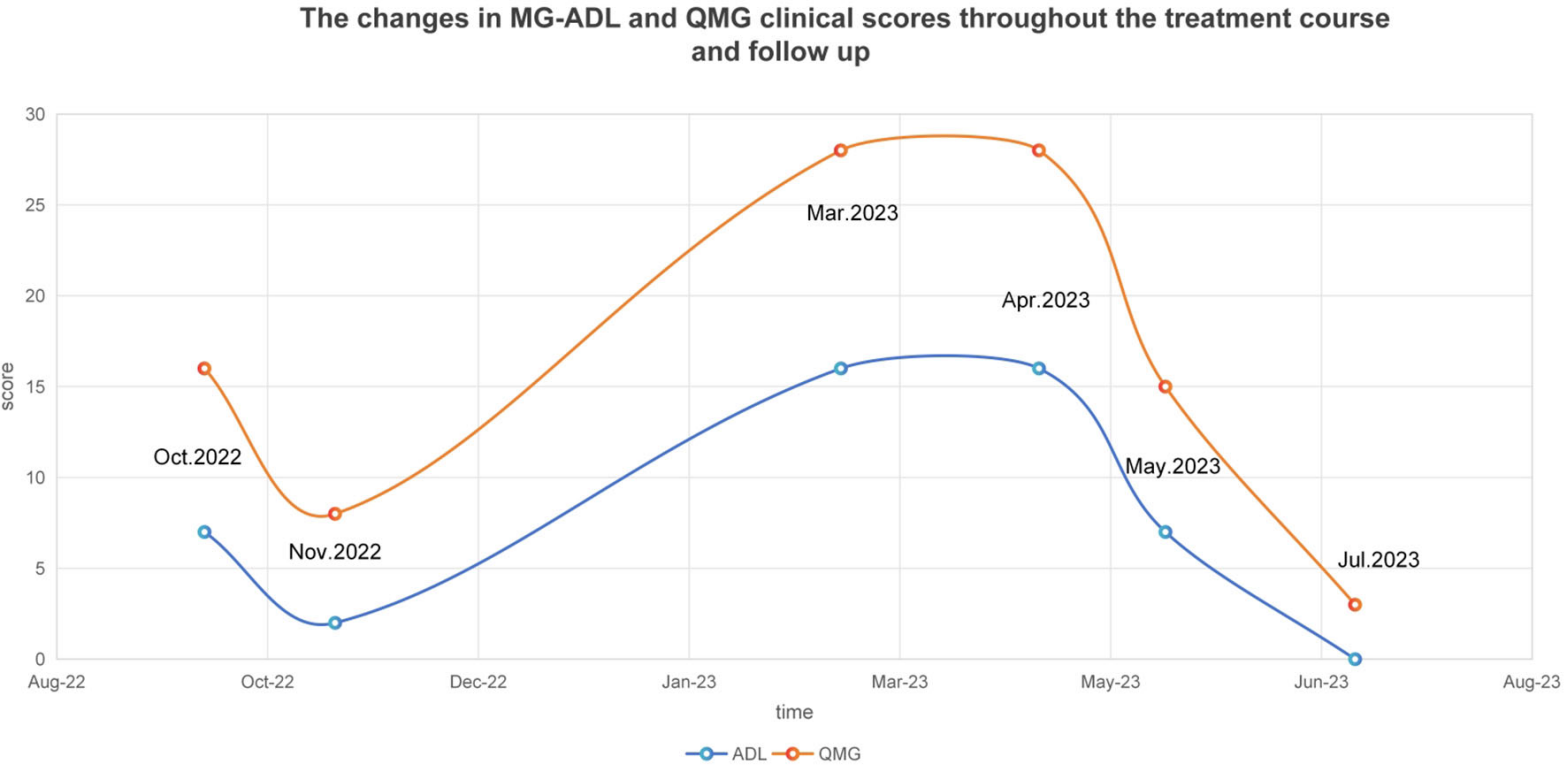
The changes in MG-ADL and OMG clinical scores throughout the treatment course and follow up.

Based on the patient’s favorable clinical response and tolerability during the induction phase, and with the aim of reducing treatment burden while maintaining long-term symptom control, an off-label maintenance strategy was adopted. A second treatment cycle of efgartigimod (10 mg/kg every two weeks) was initiated on July 10, 2024. During follow-up, serum anti-GAD65 antibodies became undetectable, whereas serum AChR antibodies remained positive (29.6 nmol/L in November 2024). As of December 2025, no further MG exacerbations, myasthenic crises, or recurrence of SPS-related stiffness and pain had been observed. **Figure 1** summarizes the timeline of disease progression, triggering events, and immunotherapeutic interventions. **Figure 2** shows the changes in MG-ADL and QMG clinical scores throughout the treatment course and follow-up.

## Discussion

This case describes the rare coexistence of SPS, MG, and type B2 thymoma, highlighting the potential role of thymic pathology in the development of multiple autoimmune neurological disorders. To date, only a limited number of cases of coexisting SPS and MG have been reported, and thymoma has been identified in a substantial proportion of these patients (7, 17–26). As summarized in **Supplementary Table 1**, thymoma appears to be a common feature in SPS–MG overlap syndrome, and several cases have demonstrated the onset or worsening of myasthenic or stiff-person symptoms following thymectomy. These observations suggest a complex relationship between thymic pathology, immune dysregulation, and neurological autoimmunity. The sequential development of SPS (low-titer positive anti-GAD65 antibodies) and MG (anti-AChR and anti-titin antibody-positive) over a four-year period in our patient supports the hypothesis that thymic neoplasms may disrupt central immune tolerance through aberrant expression of self-antigens, including AChR, thereby promoting the generation of multiple autoreactive lymphocyte clones and autoantibodies (27, 28). Notably, the serum anti-GAD65 titer of 1:100 in this patient is low and below the diagnostic threshold for SPSD. Low-titer anti-GAD65 can appear in non-neurological conditions and healthy people. Without positive CSF results or a quantitative radioimmunoassay, this finding alone isn’t diagnostic. However, low-titer anti-GAD65 antibodies, along with thymoma and anti-AChR-positive MG, might indicate broader immune dysregulation. This case highlights the importance of long-term surveillance for thymoma and the emergence of additional autoimmune neurological disorders in patients with SPS, particularly those with persistent autoimmune activity. Furthermore, this case raises the possibility that thymoma may contribute to the development of multiple autoimmune neurological syndromes beyond MG, although the precise relationship between thymoma and anti-GAD65 autoimmunity remains to be elucidated.

**Figure 3:**
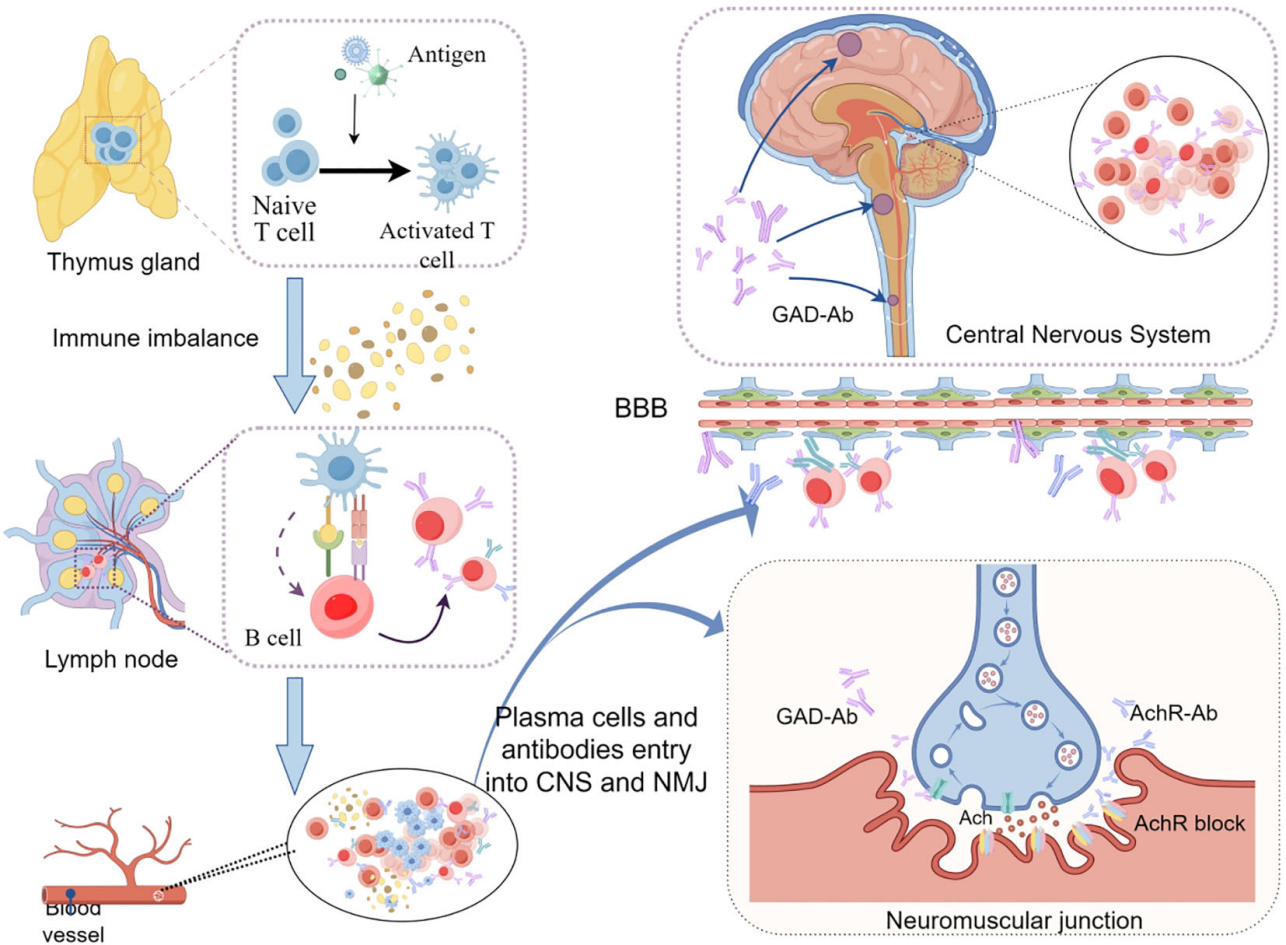
Proposed mechanism of overlapping autoimmune responses in SPS and MG.

External factors, particularly COVID-19 vaccination and subsequent SARS-CoV-2 infection, may have contributed to disease progression in our patient (29). The temporal association between the onset of MG symptoms and COVID-19 vaccination, followed by a myasthenic crisis after SARS-CoV-2 infection, is consistent with a possible “two-hit” model. In this framework, immune activation following vaccination may have unmasked pre-existing subclinical autoimmunity, whereas viral infection may have further amplified inflammatory and autoimmune responses, ultimately precipitating disease exacerbation (30). The mechanisms underlying the coexistence of SPS and MG in patients with thymoma remain poorly understood. Karsidag et al. reported a patient with SPS-MG overlap syndrome and proposed that anti-titin antibodies, which are strongly associated with thymoma-associated MG, may contribute to disease pathogenesis through molecular mimicry with neuronal antigens (31). In addition, thymoma-related defects in central immune tolerance may facilitate the generation of multiple autoreactive T- and B-cell clones, resulting in the production of distinct autoantibodies targeting both the central nervous system and neuromuscular junction Based on the clinical course of our patient and previous reports, we speculate that thymoma-induced immune dysregulation, together with peripheral immune activation triggered by environmental factors, may promote the development of overlapping autoimmune responses involving both SPS and MG. A schematic illustration of the proposed pathogenic mechanisms is shown in **Figure 3**.

Therapeutic management was challenging because of the refractory nature of the overlapping autoimmune disorders. Despite thymectomy, plasma exchange, intravenous immunoglobulin, corticosteroids, and rituximab, the patient experienced progressive MG deterioration following SARS-CoV-2 infection, ultimately developing a myasthenic crisis. Efgartigimod, a neonatal Fc receptor (FcRn) inhibitor, induced rapid and sustained clinical improvement, likely through selective reduction of pathogenic IgG autoantibodies (26, 32–34). To our knowledge, this is the first reported case of successful periodic efgartigimod treatment in a patient with coexisting SPS and MG. The favorable clinical response observed in our patient suggests that FcRn inhibition may represent a promising therapeutic strategy for refractory SPS–MG overlap syndrome, particularly in the presence of multiple pathogenic autoantibodies. Further studies are warranted to evaluate the efficacy and safety of FcRn inhibitors in larger cohorts of patients with overlapping autoimmune neurological disorders.

Long-term management of SPS–MG overlap syndrome requires a multidisciplinary approach involving neurologists, thoracic surgeons, rehabilitation specialists, and immunologists (35, 36). In our patient, relapse of SPS following corticosteroid withdrawal and subsequent progression of MG after thymoma treatment and SARS-CoV-2 infection highlight the dynamic nature of autoimmune dysregulation and the need for sustained clinical surveillance. In addition to immunotherapy, symptomatic treatments remain important for controlling stiffness, spasms, and functional impairment in patients with SPS (37). Future studies should focus on identifying predictors of disease progression and treatment response, as well as elucidating the immunopathological mechanisms linking thymoma, SPS, and MG. Improved understanding of these interactions may facilitate earlier diagnosis and the development of more targeted therapeutic strategies for patients with overlapping autoimmune neurological disorders.

## Data Availability

The original contributions presented in the study are included in the article/**Supplementary Material**. Further inquiries can be directed to the corresponding author.
